# Classification of multiple sclerosis based on patterns of CNS regional atrophy covariance

**DOI:** 10.1002/hbm.25375

**Published:** 2021-02-24

**Authors:** Charidimos Tsagkas, Katrin Parmar, Simon Pezold, Christian Barro, Mallar M. Chakravarty, Laura Gaetano, Yvonne Naegelin, Michael Amann, Athina Papadopoulou, Jens Wuerfel, Ludwig Kappos, Jens Kuhle, Till Sprenger, Cristina Granziera, Stefano Magon

**Affiliations:** ^1^ Neurologic Clinic and Policlinic, Departments of Medicine, Clinical Research and Biomedical Engineering University Hospital Basel and University of Basel Basel Switzerland; ^2^ Translational Imaging in Neurology (ThINK) Basel, Department of Medicine and Biomedical Engineering University Hospital Basel and University of Basel Basel Switzerland; ^3^ Medical Image Analysis Center AG Basel Switzerland; ^4^ Department of Biomedical Engineering University of Basel Allschwil Switzerland; ^5^ Ann Romney Center for Neurologic Diseases, Department of Neurology Brigham and Women's Hospital, Harvard Medical School Boston Massachusetts USA; ^6^ Department of Psychiatry McGill University Montreal QC Canada; ^7^ Cerebral Imaging Centre—Douglas Mental Health University Institute Verdun QC Canada; ^8^ Department of Biomedical Engineering McGill University Montreal QC Canada; ^9^ F. Hoffmann‐La Roche Ltd Basel Switzerland; ^10^ NeuroCure Clinical Research Center, Charité—Universitätsmedizin Berlin, Corporate Member of Freie Universität Berlin Humboldt‐Universität zu Berlin and Berlin Institute of Health Berlin Germany; ^11^ Department of Neurology DKD HELIOS Klinik Wiesbaden Germany; ^12^ Pharma Research and Early Development Roche Innovation Center Basel, F. Hoffmann‐La Roche Ltd. Basel Switzerland

**Keywords:** atrophy, biomarkers, classification, demyelinating autoimmune diseases, MRI, multiple sclerosis, neurodegeneration

## Abstract

There is evidence that multiple sclerosis (MS) pathology leads to distinct patterns of volume loss over time (VLOT) in different central nervous system (CNS) structures. We aimed to use such patterns to identify patient subgroups. MS patients of all classical disease phenotypes underwent annual clinical, blood, and MRI examinations over 6 years. Spinal, striatal, pallidal, thalamic, cortical, white matter, and T2‐weighted lesion volumes as well as serum neurofilament light chain (sNfL) were quantified. CNS VLOT patterns were identified using principal component analysis and patients were classified using hierarchical cluster analysis. 225 MS patients were classified into four distinct Groups A, B, C, and D including 14, 59, 141, and 11 patients, respectively). These groups did not differ in baseline demographics, disease duration, disease phenotype distribution, and lesion‐load expansion. Interestingly, Group A showed pronounced spinothalamic VLOT, Group B marked pallidal VLOT, Group C small between‐structure VLOT differences, and Group D myelocortical volume increase and pronounced white matter VLOT. Neurologic deficits were more severe and progressed faster in Group A that also had higher mean sNfL levels than all other groups. Group B experienced more frequent relapses than Group C. In conclusion, there are distinct patterns of VLOT across the CNS in MS patients, which do not overlap with clinical MS subtypes and are independent of disease duration and lesion‐load but are partially associated to sNfL levels, relapse rates, and clinical worsening. Our findings support the need for a more biologic classification of MS subtypes including volumetric and body‐fluid markers.

## Graphical abstract

In this work, we identified distinct patterns of volume loss over time in different central nervous system structures of multiple sclerosis patients. Using these patterns, we were able to robustly classify multiple sclerosis patients in separate groups. These groups did not overlap with standard clinical multiple sclerosis subtypes (relapsing‐remitting, secondary progressive and primary progressive disease) and were independent of disease duration and lesion‐load, but were partially associated with serum neurofilament light chain levels, relapse rates and clinical worsening.

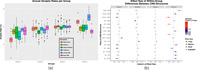

## 1 INTRODUCTION

Increasing evidence suggests that relapsing–remitting, secondary progressive and primary progressive MS patients share multiple clinical and paraclinical features, challenging the established phenotypical distinction. For example, even early relapsing–remitting MS patients demonstrate clear signs of progressive clinical worsening independent of relapses (Kappos et al., 2020). Numerous previous studies have shown contradictory results on volume loss differences over time (VLOT) in various CNS structures between clinical phenotypes (De Stefano et al., 2010; Eshaghi et al., 2018; Tsagkas et al., 2018b; Tsagkas et al., 2020). Furthermore, a recent post‐mortem study identified a separate patient group, named myelocortical MS, with demyelination restricted to the cortical gray matter and the spinal cord, which included patients from all clinical phenotypes (Trapp et al., 2018). Thus, a classification that better reflects underlying pathology is still needed and might be crucial for the effective selection of disease‐modifying treatments (Coetzee & Thompson, 2018).

In healthy adults, brain volume changes over time vary both across individuals and CNS regions (Raz et al., 2005). In previous studies, longitudinal atrophy measures have displayed a large variance between MS patients (De Stefano et al., 2010; Eshaghi et al., 2018; Tsagkas et al., 2018b). Apart from these between‐patient heterogeneities, several studies have shown dissociation in the progression of atrophy among different CNS compartments, suggesting an additional spatiotemporal within‐subject heterogeneity (Eshaghi et al., 2018; Tsagkas et al., 2018b). This indicates that volume loss, progresses independently across CNS regions. Hence, we hypothesized that the anatomic patterns of atrophy progression over time could distinguish patient groups with different underlying pathomechanisms.

In this study, we aimed to reveal the presence of distinct patterns of VLOT between different CNS‐structures and use these to identify patient subgroups in a large MS‐cohort. We then explored the clinical, radiological, and serological features of the identified patient groups.

## 2 MATERIALS AND METHODS

### 2.1 Study design and participants

Clinical and MRI data of an ongoing large‐scale cohort of MS patients (260 patients in total) from a single center (Multiple Sclerosis Center, University Hospital, Basel, Switzerland) were analyzed, retrospectively. All of the patients participating in our study have been previously reported (Magon et al., 2020; Tsagkas et al., 2018a; Tsagkas et al., 2018b; Tsagkas et al., 2020). Patients were followed for up to 6 years. The MS diagnosis was made in accordance with international panel established criteria (McDonald et al., 2001). The local ethics committee approved the study (EKBB‐46/04) and all patients provided written informed consent.

### 2.2 Procedures

All patients underwent yearly standardized neurological examinations including the Expanded Disability Status Scale (EDSS; www.neurostatus.org) by trained and certified examiners, the dominant hand and non‐dominant hand 9‐hole peg test (D9HPT and ND9HPT) as well as the timed 25‐ft walk test (T25fwt). The occurrence of new relapses was recorded at every visit. Serum samples were collected on the same day as the clinical visit and serum neurofilament light chain (sNfL) levels were measured by Simoa assay as previously described (Barro et al., 2018).

All MRI scans were performed on the same 1.5 T Magnetom Avanto MR scanner (Siemens Healthineers, Erlangen, Germany). The MRI‐protocol included a high‐resolution three‐dimensional T1‐weighted magnetization‐prepared rapid gradient‐echo (MPRAGE) sequence of the brain, acquired in sagittal orientation (TR/TI/TE = 2080/1100/3.0 ms; flip angle = 15°, 160 slices, resolution: 0.98 × 0.98 × 1 mm^3^), which also covered the upper‐cervical spinal cord. Additionally, a double echo SE proton density/T2‐weighted sequence was acquired (TR/TE1/TE2 = 3980/14/108 ms; flip angle = 180°, 40 slices, 3 mm slice thickness without gap with an in‐plane resolution of 1 × 1 mm^2^).

### 2.3 MRI analysis

#### 2.3.1 Lesion segmentation

All brain white matter lesions were segmented on T2‐weighted and proton density images by trained expert observers according to the standard operating procedures used at the Medical Image Analysis Center for the analysis of clinical phase II and phase III trials (Magon et al., 2014). T2‐weighted lesion volume (T2LV) was calculated for the whole brain.

#### 2.3.2 Volumetry of CNS structures

All morphological analyses were performed on the T1‐weighted MPRAGE brain images.

Brain white matter was computed with the fully automated tool SIENAX (version 2.6) (Smith et al., 2004). Cortical gray matter was fully automatically segmented using CIVET (version 2.1.0) (Zijdenbos, Forghani, & Evans, 2002), as also used previously in the context of several longitudinal studies (Bhagwat et al., 2018; Makowski et al., 2016; Redolfi et al., 2015; Tsagkas et al., 2020). Before segmentation with CIVET, the T1‐weighted images were linearly registered to the standard stereotaxic space defined by the MNI ICBM 152 model (Mazziotta et al., 2001). The images were then corrected for intensity nonuniformity using N3 (Sled et al., 1998) and a linear registration to the model (Collins et al., 1994) was applied.

The volume of the deep gray matter nuclei including thalamus, striatum, and globus pallidus was estimated based on an established nomenclature using MAGeT as previously described (Chakravarty et al., 2013) and deployed in previous cross‐sectional studies (Magon et al., 2015). Data pre‐processing and detailed MAGeT application was described in a previous study (Magon, Chakravarty, et al., 2014). In short, both the medical imaging netCDF (MINC) toolkit (version 2) and the advance normalization tools (ANTs) were deployed as follows: (1) bias field correction using N4‐correction algorithm (Tustison et al., 2010); (2) non‐local means denoising (Manjón et al., 2010); (3) affine registration using a normalized mutual information objective function (Studholme et al., 2001); and (4) brain extraction using the BEaST algorithm (Eskildsen et al., 2012). The preprocessed data were then used as input for the MAGeT algorithm. The MAGeT registration procedure is also similar to CIVET, which should at least reduce potential biases between these two algorithms that calculated the cortical, thalamic, pallidal, and striatal volumes used in this study. Quality assessment was performed on each T1‐weighted image by a trained researcher to ensure segmentation correctness.

MAGeT, CIVET, and SIENAX were performed on T1‐weighted images after lesion filling using the approach previously proposed by our group (Magon et al., 2014) in order to reduce biases related to tissue misclassification and improve the registration step (Sdika & Pelletier, 2009). The SIENAX baseline volume‐correction factor was used for normalizing cortical gray matter, white matter, and deep gray matter nuclei regarding variations of head size. All analyses were performed on these corrected volumes.

Spinal cord volume analysis was performed using the established CORDIAL semi‐automatic software, as described in previous methodological and clinical studies (Amann et al., 2016; Tsagkas et al., 2018b). Shortly, the segmentation was carried out over a 35 mm long spinal cord segment, starting 27 mm below the cisterna pontis, which corresponds roughly to the spinal cord volume between the Foramen magnum and the C2/C3 intervertebral disk. Segmentations were visually inspected for quality and excluded from further statistical analysis in case of segmentation errors.

### 2.4 Statistical analysis

#### 2.4.1 Clustering of MS patients

The mean annual loss rate of cortical gray matter, brain white matter, thalamus, striatum, globus pallidus, and the spinal cord over a maximum follow‐up time of up to 6 years was determined as the average percentage of the annualized changes between all available time‐points for every patient. In order to correct for the effect of age on the mean annual atrophy rates of those regions, we calculated the residuals from a regression analysis between the atrophy rate of each region and age. Subsequently, a principal component analysis was performed on these residuals of the mean annual loss rates using the singular value decomposition method (Husson, Josse, & Pages, 2010). Principal components represent different parts of the between‐structure covariance and, thus, reflect between‐structure patterns of CNS VLOT. Each principal component extracted using this approach highlighted the difference or similarity between volume changes across individual CNS regions.

**FIGURE 1:**
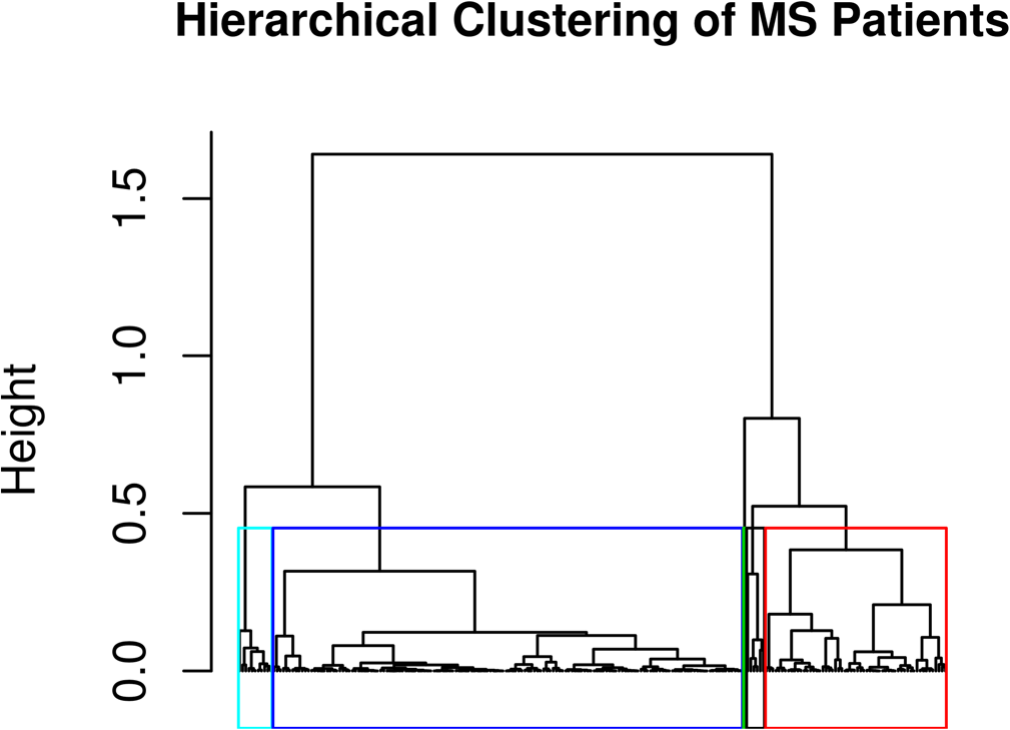
Dendrogram of hierarchical clustering of multiple sclerosis patients using Ward's criterion. Y‐axis displays the observations' height, which is a measure of proximity of either individual data points and/or clusters. The turquoise box contains all patients classified in Group A. The red box contains all patients classified in Group B. The blue box contains all patients classified in Group C. The black box contains all patients classified in Group D. The green line signifies the single outlier excluded from further analysis

Principal components kept in further statistical analysis had to meet the following criteria: (1) >90% of total variance retained, (2) eigenvalue >0.7, (3) “broken stick rule,” (4) scree plot “elbow rule” (Jolliffe, 2002). Hierarchical clustering was then performed using Ward's‐criterion on the selected principal components (Husson et al., 2010). Extraction of the resulting groups was done after visualizing the cluster dendrogram (Figure 1). In order to validate the stability of the resulting clusters, we repeated the principal component analysis and subsequently the hierarchical clustering analysis 10,000‐fold, using the same methods described above. In every repetition, a contribution weight of 0.0001 to the principal component analysis was ascribed to a random 10% of our patients and a contribution weight of 2 to another random 10%, leaving the rest 80% with a uniform contribution weight of 1. This allowed “suppression” of a smaller subject number—thus, avoiding bias against smaller clusters—but inserted significant data modifications for reliable cross‐validation of our classification. After each repetition, the adjusted Rand index (Rand, 1971) and Meila's variation of information distance (Meilă, 2007) were calculated to measure the similarity between the new clustering outcome and our initial classification.

#### 2.4.2 Group comparisons

In order to approximate a normal distribution, logarithmic transformations were performed for the EDSS, D9HPT and ND9HPT, whereas an inverse transformation was performed for the T25fwt. Baseline MS severity score (Roxburgh et al., 2005), mean annual relapse number and the mean sNfL concentration were calculated. Comparisons of baseline demographic factors, clinical measurements, and number of follow‐ups between groups were made using Welch's two sample *t*‐tests and Pearson's chi‐squared test with Yates's continuity correction. Between‐group differences in regard to baseline MRI measures and annual rates of VLOT were performed using analyses of covariance, while correcting for age, sex, and disease duration.

Statistical analysis of the patients' MRI metrics and clinical outcomes (EDSS, D9HPT, ND9HPT, T25fwt) over 6 years was performed using separate linear mixed effect models (LMER) in order to explore between‐group differences. For both model types, this was done using a random intercept and a random time‐slope for each subject to allow for within‐subject and between‐subject variance. Independent variables were entered blockwise keeping the following sequence: first demographics, then disease duration and finally the aforementioned classification result. Each variable was tested both for its correlation to the intercept as well as to the slope over time of the dependent variable. All independent variables without statistical significance were excluded from the final model. Within‐group comparisons between CNS structures were performed using paired sample *t*‐tests, whereas respective effect sizes were computed as Cohen's *d* for paired samples.

We performed a Cox regression analysis in a backwards stepwise fashion including sex, age, disease duration, and the classification outcome of our analysis to evaluate differences between the identified MS patient groups in terms of disease progression and time‐to‐disease progression. Clinical disease progression was defined according to the following conventions: (1a) an increase of 1 point in the EDSS if the baseline EDSS score was ≤5.5 or (1b) an increase of 0.5 point in the EDSS if the baseline EDSS score was >5.5, and (2) no relapse in the last 12 months.

## 3 RESULTS

### 3.1 Results of clustering analysis

After excluding MRI sessions with segmentation failures or errors (162 of 1,271 MRI) in either of the quantified CNS regions and patients with availability of only 1 MRI dataset (in total, 29 patients excluded due to missing values) as well as patients diagnosed with clinically isolated syndrome that did not convert to clinically definite MS during follow‐up (5 patients in total), a total of 226 patients, and 1,080 examination datasets (including clinical, sNfL, and MRI data) were entered in the statistical analysis.

In a first step, we performed a regression analysis between the annual atrophy rates of the spinal cord, striatum, globus pallidus, thalamus, cortical gray matter, and brain white matter on one side and age on the other. Subsequently, we performed a principal component analysis using the residuals from each regression analysis. The first four principal components accounted cumulatively for 92% of the total data‐variance and were kept in further statistical analysis. Table S1 displays Pearson's correlation coefficients between the residuals of the mean annual atrophy rates of the analyzed CNS regions after correcting for age. Detailed results of those four principal components are displayed in Table 1.

**TABLE 1:** Results of principal component analysis of multiple sclerosis patients

	PC1	PC2	PC3	PC4
Total data variance (%)	38.9	26.1	15.8	11.2
Eigenvalue	2.69	1.81	1.09	0.77
SC				
*Coordinate*	0.95	−0.16	−0.76	−0.48
*QoVR*	0.52	0.02	0.33	0.13
*Contribution (%)*	29.5	1.29	39.6	29.3
Striatum				
*Coordinate*	0.43	0.43	−0.01	0.23
*QoVR*	0.31	0.30	0.00	0.09
*Contribution (%)*	6.13	8.65	0.00	6.97
Globus Pallidus				
*Coordinate*	0.53	0.77	0.77	−0.42
*QoVR*	0.18	0.39	0.03	0.12
*Contribution (%)*	9.03	27.9	30.4	22.8
Thalamus				
*Coordinate*	0.80	0.13	−0.22	0.52
*QoVR*	0.58	0.02	0.04	0.24
*Contribution (%)*	20.9	0.85	3.27	34.9
Cortical GM				
*Coordinate*	1.02	−0.65	0.58	0.13
*QoVR*	0.55	0.23	0.18	0.01
*Contribution (%)*	33.8	20.7	22.6	2.17
Brain WM				
*Coordinate*	0.14	0.93	−0.24	0.17
*QoVR*	0.02	0.75	0.05	0.03
*Contribution (%)*	0.68	40.6	4.06	3.88

Abbreviations: GM, gray matter; PC, principal component; QoVR, quality of variable representation; SC, spinal cord; WM, white matter.

The dendrogram of the hierarchical clustering is shown in Figure 1. 225 out of 226 patients were classified in four different groups, which are from now on referred to as Groups A (14 patients), B (59 patients), C (141 patients), and D (11 patients). One patient (male, baseline age 21 years, baseline disease duration 10 years, relapsing–remitting MS of pediatric onset) was not classified in any of these four groups and was therefore excluded from further analysis as an outlier. The groups' demographic and baseline clinical and volumetric characteristics are displayed in Table 2. Groups did not differ in age, sex, disease duration, and clinical phenotype distribution. Patients of Group A had a higher number of untreated patients compared to Group C, whereas the distribution of treatments used in Group C was different compared to Group D.

**TABLE 2:** Demographics and baseline clinical and volumetric characteristics of patients with MS per group

Characteristics	Group A	Group B	Group C	Group D	*p*‐value
Number of patients	14	59	141	11	
Baseline age (years)					n.s.
*Mean ± SD*	40.6 ± 8.83	45.6 ± 11.2	44.5 ± 10.7	46.7 ± 13.8	
*Range*	30–56	21–67	19–65	22–66	
Sex (female/male)	7 / 7	36 / 23	97 / 44	10/1	n.s.
Clinical classification	8 RR, 3 SP, 3 PP	39 RR, 13 SP, 1 PP, 4 RR → SP, 2 CIS → RR	102 RR, 21 SP, 7 PP, 10 RR → SP, 1 CIS → RR	7 RR, 4 SP	n.s.
Baseline disease duration (y)					n.s.
*Mean ± SD*	10.6 ± 8.26	12.4 ± 9.89	13.4 ± 8.89	14.1 ± 3.81	
*Range*	1–30	0–37	0–47	7–19	
Baseline MSSS					A vs. B: * A vs. C: *** A vs. D: *
*Mean ± SD*	6.15 ± 2.24	4.53 ± 2.02	3.79 ± 2.03	4.04 ± 2.17	
*Range*	1.18–9.70	0.70–8.32	0.21–7.81	1.54–7.34	
Baseline EDSS					A vs. C: *
*Median*	3.75	3.0	2.5	3.0	
*Range*	1.0–7.5	0.0–6.5	0–6.5	1.5–6.0	
Baseline T25fwt (s)					n.s.
*Mean ± SD*	8.35 ± 7.01	8.55 ± 13.9	6.61 ± 7.84	7.97 ± 5.54	
*Range*	3.65–28.2	3.25–93.3	2.15–82.7	3.50–22.3	
Baseline D9HPT (s)					n.s.
*Median*	25.6 ± 9.08	23.4 ± 7.73	21.0 ± 5.51	23.2 ± 6.57	
*Range*	16.0–49.3	13.5–47.0	14.5–53.3	16.3–34.4	
Baseline ND9HPT (s)					A vs. B: *** A vs. C: *** A vs. D: *
*Median*	32.6 ± 13.0	24.1 ± 8.7	22.2 ± 7.00	24.0 ± 5.85	
*Range*	18.8–65.2	15.7–71.6	14.5–80.0	16.0–32.6	
Treatment (treated/ untreated)	4 / 10	38 / 21	96 / 45	8 / 3	A vs. C: *
*Azathioprin*	—	—	5	—	C vs. D: **
*Interferon‐1β/1a*	3	32	73	6	
*Glatiramer‐acetate*	1	7	19	—	
*Mitoxantrone*	—	1	3	2	
Number of follow‐ups					A vs. B: * A vs. C: ** B vs. C: *** B vs. D: ** C vs. D: ***
*Mean ± SD*	3.00 ± 1.18	4.19 ± 1.82	5.34 ± 1.43	2.64 ± 0.81	
*Range*	2–6	2–7	2–7	2–6	
Maximum follow‐up time (years)					A vs. B: * A vs. C: *** B vs. C: *** B vs. D: ** C vs. D: ***
*Mean ± SD*	2.29 ± 1.59	3.76 ± 2.08	4.94 ± 1.38	1.91 ± 1.22	
*Range*	1–6	1–6	1–6	1–6	
Baseline SC volume (cm^3^)					n.s.
*Mean ± SD*	2.41 ± 0.28	2.40 ± 0.30	2.38 ± 0.33	2.18 ± 0.29	
*Range*	1.89–2.89	1.70–3.05	1.61–3.07	1.75–2.58	
Baseline striatum volume (cm^3^)					n.s.
*Mean ± SD*	20.0 ± 1.87	20.9 ± 2.48	21.0 ± 2.40	20.3 ± 2.22	
*Range (min; max)*	16.7–23.5	15.9–26.7	15.1–29.0	17.3–24.7	
Baseline Globus Pallidus volume (cm^3^)					n.s.
*Mean ± SD*	3.01 ± 0.37	3.18 ± 0.34	3.24 ± 0.36	3.17 ± 0.29	
*Range (min; max)*	2.31–3.66	2.45–4.07	2.35–4.60	2.65–3.57	
Baseline thalamus volume (cm^3^)					A vs. C: *
*Mean ± SD*	11.9 ± 1.89	13.0 ± 1.86	13.2 ± 2.04	12.3 ± 2.47	
*Range (min; max)*	8.33–14.5	8.55–17.1	7.35–17.3	9.20–17.5	
Baseline cortical GM volume (cm^3^)					n.s.
*Mean ± SD*	650.5 ± 34.5	652.2 ± 60.7	644.2 ± 54.0	620.5 ± 47.7	
*Range (min; max)*	591.8–731.6	529.9–781.2	513.7–793.6	528.3–701.2	
Baseline brain WM volume (cm^3^)					n.s.
*Mean ± SD*	727.9 ± 42.4	730.9 ± 47.4	735.5 ± 51.2	730.3 ± 52.2	
*Range (min; max)*	644.2–806.5	596.4–831.7	603.2–844.7	528.2–701.2	

*Note*: Between‐group comparisons were performed using Welch's two sample *t*‐test and Pearson's chi‐squared test with Yate's continuity or Bonferroni's correction where appropriate. Comparisons of baseline volumes between groups were performed with analysis of covariance (ANCOVA), after correcting for sex, age, and disease duration.

Abbreviations: CIS → RR, clinically isolated syndrome transitioned to relapsing remitting multiple sclerosis; D9HPT, dominant hand 9‐hole peg test; EDSS, expanded disability status scale; GM, gray matter; MSSS, multiple sclerosis severity score; n.s., not significant for any pairwise comparisons between Groups A, B, and C; ND9HPT, non‐dominant hand 9‐hole peg test; PP, primary progressive; RR, relapsing remitting; RR → SP, relapsing remitting transitioned to secondary progressive multiple sclerosis; SC, spinal cord; SD, standard deviation; SP, secondary progressive; T25fwt, timed 25‐foot walk test; WM, white matter.

Cross‐validation of the clustering process with 10,000‐fold repetition showed a clustering agreement with a mean adjusted Rand index of 0.72 ± 0.17 (with 0 corresponding to random clustering results and 1 to absolute agreement between clustering outcomes) and a Meila's variation of information distance of 0.57 ± 0.30 (with 0 corresponding to absolute agreement and 2.35—equal to log(225)—corresponding to absolute disagreement between clustering outcomes).

### 3.2 Within‐group patterns of VLOT

**FIGURE 2:**
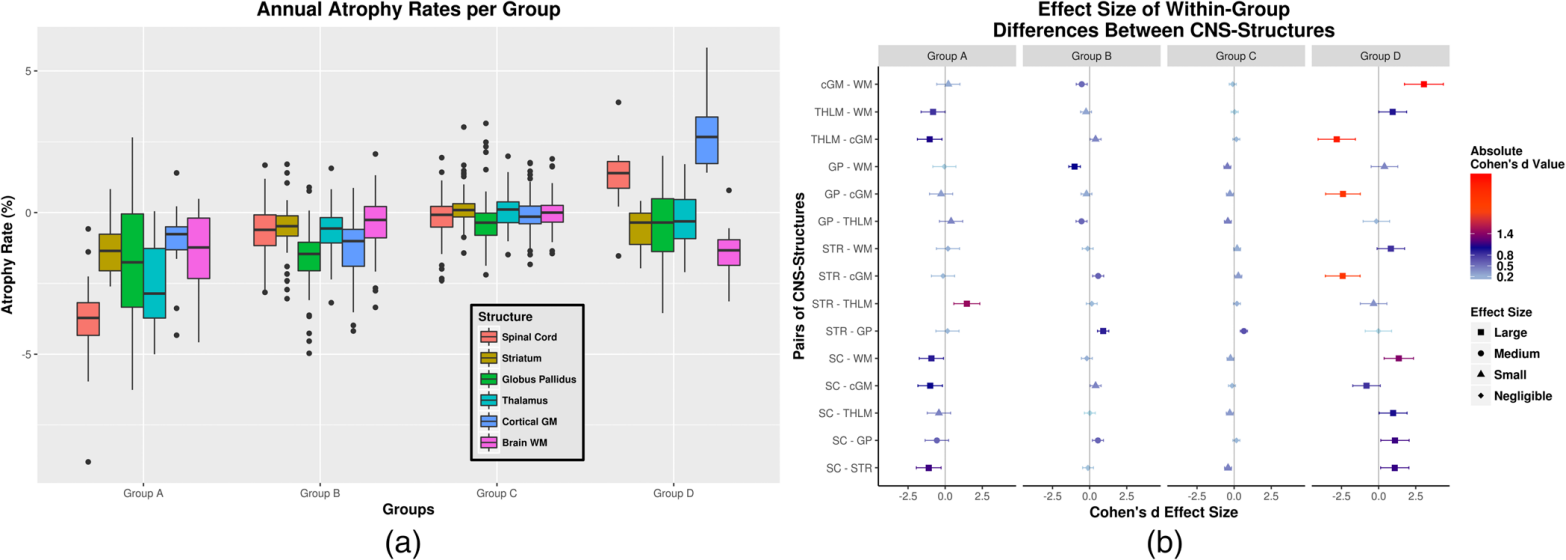
(a) Boxplots of mean annual volume changes of the spinal cord, striatum, globus pallidus, thalamus, cortical gray matter (GM), and brain white matter (WM) by group. Whiskers correspond to annual volume changes' 25th and 75th percentiles. (b) Effect size of within‐group differences in volume loss over time between CNS‐structures by group. Whiskers correspond to the effect size 95% confidence intervals. Absolute Cohen's *d* values are indicated with a light blue to red gradient. Shape indicates negligible, small, medium, and large effect sizes. Abbreviations: SC, spinal cord; STR, striatum; GP, globus pallidus; THLM, thalamus; cGM, cortical gray matter; WM, brain white matter

Mean annual atrophy rates of the spinal cord, striatum, globus pallidus, thalamus, cortical gray matter, and brain white matter volumes as well as within‐group differences between the atrophy rates of all CNS‐structures and the respective size effects are reported in Table 3 and Figure 2. Group A showed a more prominent spinal cord (−3.82%/year) and thalamic (−2.65%/year) VLOT compared to all other CNS‐structures (absolute Cohen's *d* of spinal cord and thalamus compared to all other structures: 0.56–1.11 and 0.40–1.46). Group B was characterized by a more prominent pallidal (−0.93%/year) VLOT compared to all other structures (absolute Cohen's *d* between globus pallidus and all other structures: 0.20–1.02). Between‐structure differences of VLOT were generally rather small in Group C. Finally, Group D demonstrated an increase of spinal cord (1.29%/year) and cortical gray matter (2.82%/year) volume over time and a more prominent brain white matter VLOT (−1.37%/year) (absolute Cohen's *d* of cortical gray matter, spinal cord and brain white matter compared to all other structures: 0.39–3.05).

**TABLE 3:** Annual volume changes as well as within‐group differences and effect size of within‐group differences of MRI measures in patients with multiple sclerosis

Annual volume changes of MRI measures	Group A *n = 14*	Group B *n = 59*	Group C *n = 141*	Group D *n = 11*	Between‐group differences *p*‐value
SC (%/years)					A vs. B: *** A vs. C: *** A vs. D: *** B vs. C: ** B vs. D: *** C vs. D: ***
*Mean ± SD*	−3.82 ± 1.97	−0.67 ± 0.97	−0.18 ± 0.66	1.29 ± 1.32	
*Range (min; max)*	−8.81; −0.58	−2.81; 1.68	−2.40; 1.94	−1.53; 3.90	
Striatum (%/years)					A vs. B: ** A vs. C: *** B vs. C: *** C vs. D: **
*Mean ± SD*	−1.27 ± 1.04	−0.54 ± 0.84	0.15 ± 0.53	−0.58 ± 0.80	
*Range (min; max)*	−2.60; 0.83	−3.03; 1.71	−1.43; 3.03	−1.97; 0.42	
GP (%/years)					A vs. C: *** A vs. D: * B vs. C: *** B vs. D: **
*Mean ± SD*	−1.65 ± 2.37	−1.61 ± 1.09	−0.33 ± 0.79	−0.55 ± 1.50	
*Range (min; max)*	−6.26; 2.66	−4.97; 0.89	−2.20; 3.16	−3.54; 2.01	
Thalamus (%/years)					A vs. B: *** A vs. C: *** A vs. D: *** B vs. C: ***
*Mean ± SD*	−2.65 ± 1.59	−0.67 ± 0.87	0.05 ± 0.57	−0.28 ± 1.10	
*Range (min; max)*	−5.00; 0.05	−3.18; 1.57	−1.49; 1.99	−2.11; 1.71	
Cortical GM (%/years)					A vs. C: *** A vs. D: *** B vs. C: *** B vs. D: *** C vs. D: ***
*Mean ± SD*	−1.03 ± 1.42	−1.28 ± 1.05	−0.06 ± 0.61	2.82 ± 1.41	
*Range (min; max)*	−4.33; 1.40	−4.18; 0.87	−1.83; 1.77	1.41; 5.83	
Brain WM (%/years)					A vs. B: *** A vs. C: *** B vs. C: ** B vs. D: ** C vs. D: ***
*Mean ± SD*	−1.50 ± 1.61	−0.40 ± 1.01	0.03 ± 0.54	−1.37 ± 1.02	
*Range (min; max)*	−4.58; 0.49	−3.34; 2.07	−1.45; 1.90	−3.13; 0.79	
Within‐group differences between annual volume changes of MRI‐measures	Group A *n = 14*	Group B *n = 59*	Group C *n = 141*	Group D *n = 11*	Within‐group differences *p*‐value
Cortical GM–Brain WM					B:*** D:***
*Mean differences (%)* *[95% CI (min; max)]*	0.47 [−0.80; 1.73]	−0.88 [−1.31; −0.45]	−0.08 [−0.23; 0.06]	4.18 [3.26; 5.11]	
*Mean Cohen's d* *[95% CI (min; max)]*	0.21 [−0.57; 0.99]	−0.53 [−0.91; −0.16]	−0.09 [−0.33; 0.14]	3.05 [1.74; 4.36]	
Thalamus–Brain WM					A:** D:*
*Mean differences (%)* *[95% CI (min; max)]*	−1.15 [−1.97; −0.33]	−0.30 [−0.61; 0.02]	0.02 [−0.08; 0.13]	1.09 [0.32; 1.86]	
*Mean Cohen's d* *[95% CI (min; max)]*	−0.81 [−1.62; −0.01]	−0.24 [−0.61; 0.12]	0.04 [−0.20; 0.27]	0.95 [0.01; 1.89]	
Thalamus–Cortical GM					A:** B:** D:***
*Mean differences (%)* *[95% CI (min; max)]*	−1.62 [−2.51; −0.72]	0.58 [0.20; 0.97]	0.11 [−0.01; 0.23]	−3.10 [−3.83; −2.36]	
*Mean Cohen's d* *[95% CI (min; max)]*	−1.04 [−1.87; −0.22]	0.40 [0.03; 0.77]	0.15 [−0.09; 0.39]	−2.82 [−4.10; −1.57]	
GP–Brain WM					B:*** C:*** D:*
*Mean differences (%)* *[95% CI (min; max)]*	−0.15 [−1.78; 1.48]	−1.21 [−1.52; −0.90]	−0.36 [−0.49–0.23]	0.81 [−0.58; 2.20]	
*Mean Cohen's d* *[95% CI (min; max)]*	−0.05 [−0.83; 0.72]	−1.02 [−1.40; −0.63]	−0.46 [−0.69; −0.22]	0.39 [−0.51; 1.29]	
GP–Cortical GM					C: *** D:***
*Mean differences (%)* *[95% CI (min; max)]*	−0.62 [−1.91; 0.67]	−0.33 [−0.73; 0.07]	−0.28 [−0.44; −0.12]	−3.37 [−4.31; −2.43]	
*Mean Cohen's d* *[95% CI (min; max)]*	−0.28 [−1.05; 0.50]	−0.21 [−0.58; 0.15]	−0.29 [−0.52; −0.05]	−2.40 [−3.57; −1.24]	
GP–Thalamus					B:*** C:***
*Mean differences (%)* *[95% CI (min; max)]*	1.00 [−0.45; 2.46]	−0.91 [−1.35; −0.48]	−0.39 [−0.54; −0.23]	−0.27 [−1.48; 0.94]	
*Mean Cohen's d* *[95% CI (min; max)]*	0.40 [−0.39; 1.18]	−0.55 [−0.92; 0.18]	−0.42 [−0.66; −0.19]	−0.15 [−1.04; 0.74]	
Striatum–Brain WM					C:* D:*
*Mean differences (%)* *[95% CI (min; max)]*	−0.23 [−0.49; 0.96]	−0.14 [−0.41; 0.13]	0.12 [0.02; 0.21]	0.79 [0.14; 1.43]	
*Mean Cohen's d* *[95% CI (min; max)]*	−0.19 [−0.59; 0.97]	−0.13 [−0.50; 0.23]	0.20 [−0.03; 0.44]	0.82 [−0.10; 1.75]	
Striatum–Cortical GM					B:*** C:*** D:***
*Mean differences (%)* *[95% CI (min; max)]*	−0.23 [−1.16; 0.70]	0.74 [0.41; 1.08]	0.20 [0.08; 0.32]	−3.40 [−4.34; −2.45]	
*Mean Cohen's d* *[95% CI (min; max)]*	−0.14 [−0.92; 0.63]	0.58 [0.20; 0.95]	0.28 [0.05; 0.52]	−2.42 [−3.59; −1.25]	
Striatum–Thalamus					A: *** C:*
*Mean differences (%)* *[95% CI (min; max)]*	1.39 [0.84; 1.94]	0.16 [−0.13; 0.45]	0.09 [0.01; 0.17]	−0.30 [−0.89; 0.29]	
*Mean Cohen's d* *[95% CI (min; max)]*	1.46 [0.59; 2.33]	0.14 [−0.22; 0.51]	0.18 [−0.05; 0.42]	−0.34 [−1.23; 0.56]	
Striatum–GP					B:*** C:***
*Mean differences (%)* *[95% CI (min; max)]*	0.38 [−1.05; 1.82]	1.07 [0.76; 1.38]	0.48 [0.36; 0.60]	−0.03 [−1.29; 1.24]	
*Mean Cohen's d* *[95% CI (min; max)]*	0.15 [−0.62; 0.93]	0.91 [0.53; 1.29]	0.66 [0.42; 0.90]	−0.01 [−0.90; 0.88]	
SC–Brain WM					A: ** C:** D: **
*Mean differences (%)* *[95% CI (min; max)]*	−2.32 [−3.76; −0.88]	−1.28 [−1.67; 0.11]	−0.20 [−0.34; −0.07]	2.65 [1.34; 3.96]	
*Mean Cohen's d* *[95% CI (min; max)]*	−0.93 [−1.75; 0.11]	−0.19 [−0.56; 0.18]	−0.25 [−0.48; −0.01]	1.36 [0.37; 2.35]	
SC–Cortical GM					A:** B: ** D:*
*Mean differences (%)* *[95% CI (min; max)]*	−2.79 [−4.38; −1.20]	0.60 [0.21; 0.99]	−0.12 [−0.26; 0.02]	−1.53 [−2.79; 0.27]	
*Mean Cohen's d* *[95% CI (min; max)]*	−1.01 [−1.84; −0.19]	0.40 [0.03; 0.77]	0.14 [−0.38; 0.09]	−0.81 [−1.74; 0.11]	
SC–Thalamus					C:*** D:**
*Mean differences (%)* *[95% CI (min; max)]*	−1.17 [−2.77; 0.44]	0.01 [−0.31; −0.34]	−0.23 [−0.36; −0.10]	1.57 [0.49; 2.65]	
*Mean Cohen's d* *[95% CI (min; max)]*	−0.42 [−1.20; 0.36]	0.01 [−0.35; 0.38]	−0.29 [−0.53; −0.06]	0.98 [0.04; 1.92]	
SC–GP					B:*** D:**
*Mean differences (%)* *[95% CI (min; max)]*	−2.17 [−4.41; 0.07]	0.93 [0.49; 1.36]	0.16 [−0.02; 0.33]	1.84 [0.72; 2.96]	
*Mean Cohen's d* *[95% CI (min; max)]*	−0.56 [−1.35; 0.23]	0.56 [0.18; 0.93]	0.15 [0.08; 0.39]	1.10 [0.14; 2.06]	
SC–Striatum					A: ** C: *** D: **
*Mean differences (%)* *[95% CI (min; max)]*	−2.56 [−3.89; −1.22]	−0.14 [−0.49; 0.20]	−0.32 [−0.45; −0.19]	1.87 [0.71; 3.02]	
*Mean Cohen's d* *[95% CI (min; max)]*	−1.11 [−1.94; −0.27]	−0.11 [−0.47; 0.26]	−0.41 [−0.65; −0.17]	1.09 [0.13; 2.04]	

*Note*: Comparisons of annual volume changes between groups were performed with analysis of covariance (ANCOVA), after correcting for sex, age, disease duration, and baseline volume. Within‐group comparisons were performed using paired sample *t*‐tests. Effect sizes were computed as Cohen's *d* for paired samples.

Abbreviations: GM, gray matter; GP, globus pallidus; *n*, number of subjects; n.s. = not significant for any pairwise comparisons between Groups A, B, and C or between CNS‐structures within‐groups; SC, spinal cord; SD, standard deviation; WM, white matter.

### 3.3 Longitudinal analysis of MRI measurements between groups

Detailed results for each analyzed CNS structure and T2LV are displayed in Table 4. Summarizing the most important results, Group A demonstrated significantly greater spinal cord and thalamic VLOT compared to all other groups. Group B showed significantly greater pallidal VLOT compared to groups C and D. Group C showed lower VLOT in all CNS structures compared to Groups A and B. Group D showed a significant cortical gray matter volume increase compared to all other groups and a significant spinal cord volume increase compared to Groups A and B; additionally, a significantly greater white matter VLOT than in Group C was shown. T2LV increase over time did not differ between groups.

**TABLE 4:** Average CNS volumes and volume changes over time as well as between‐group differences in patients with multiple sclerosis as calculated by linear mixed effect regression models over a maximum of 6 years

Volume of CNS structures	Group A *n = 14*	Group B *n = 59*	Group C *n = 141*	Group D *n = 11*	Between‐group differences *p*‐value
SC volume *(mm* ^*3*^ *)*					
*Average* ± *SE*	2,194 ± 77.2	2,376 ± 37.7	2,407 ± 25.2	2,327 ± 88.7	A vs. C: *
*Change per Year* ± *SE*	−81.6 ± 6.63	−15.8 ± 2.31	−3.86 ± 1.36	16.8 ± 8.15	A vs. B: *** A vs. C: *** A vs. D: *** B vs. C: *** B vs. D: ***
Striatum volume *(mm* ^*3*^ *)*					
*Average* ± *SE*	18,982 ± 535	20,720 ± 261	20,851 ± 175	19,973 ± 614	A vs. B: * A vs. C: **
*Change per Year* ± *SE*	−204 ± 37.9	−67.3 ± 12.6	20.8 ± 6.92	−92.5 ± 46.7	A vs. B: ** A vs. C: *** B vs. C: ***
GP volume *(mm* ^*3*^ *)*					
*Average* ± *SE*	2,923 ± 88.6	3,067 ± 43.0	3,182 ± 28.9	3,090 ± 102	A vs. C: *
*Change per Year* ± *SE*	−44.9 ± 8.51	−39.3 ± 2.59	−14.5 ± 1.41	−10.1 ± 10.9	A vs. C: *** B vs. C: *** B vs. D: *
Thalamus volume *(mm* ^*3*^ *)*					
*Average* ± *SE*	11,003 ± 465	12,608 ± 227	12,985 ± 152	11,894 ± 534	A vs. B: * A vs. C: ***
*Change per Year* ± *SE*	−219 ± 31.6	−84.1 ± 11.0	4.42 ± 6.14	−61.3 ± 38.5	A vs. B: *** A vs. C: *** A vs. D: ** B vs. C: ***
Cortical GM volume *(cm* ^*3*^ *)*					
*Average* ± *SE*	620 ± 11.6	638 ± 5.60	639 ± 3.73	638 ± 13.5	n.s.
*Change per Year* ± *SE*	−8.34 ± 1.72	−4.47 ± 0.53	−0.91 ± 0.30	7.42 ± 2.21	A vs. C: *** A vs. D: ** B vs. C: *** B vs. D: *** C vs. D: **
Brain WM volume *(cm* ^*3*^ *)*					
*Average* ± *SE*	705 ± 12.2	722 ± 5.91	734 ± 3.95	717 ± 14.1	n.s.
*Change per Year* ± *SE*	−5.20 ± 1.63	−2.71 ± 0.52	−0.001 ± 0.29	−7.08 ± 2.04	A vs. C: ** B vs. C: *** C vs. D: **
*T2 lesion volume (mm* ^*3*^ *)*					
*Average* ± *SE*	11,522 ± 1,681	5,313 ± 818	5,634 ± 539	8,693 ± 1889	A vs. B: ** A vs. C: **
*Change per Year* ± *SE*	510 ± 195	184 ± 68.6	32.5 ± 38.3	92.5 ± 227	n.s.

*Note*: All depicted values have been calculated using linear mixed effect regression models after correcting for sex, age, and disease duration. Comparisons between groups with regard to average volumes changes and volume changes over time were performed with post hoc analysis using the Tukey test.

Abbreviations: GM, gray matter; GP, globus pallidus; *n*, number of subjects; n.s., not significant for any pairwise comparisons between Groups A, B, and C or between CNS‐structures within‐groups; SC, spinal cord; SE, standard error; WM, white matter.

### 3.4 Longitudinal analysis of clinical outcomes between groups

**FIGURE 3:**
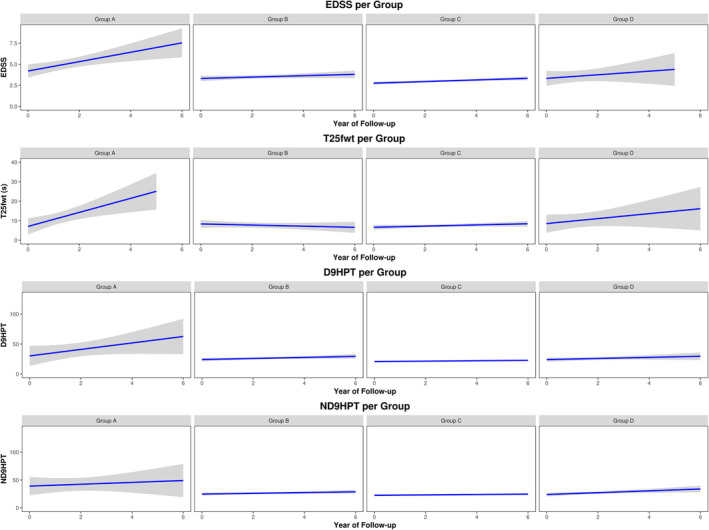
Trends of the expanded disability status scale (EDSS), Timed 25‐ft walk test (T25fwt), dominant hand and non‐dominant hand 9‐Hole Peg Test (D9HPT and ND9HPT, respectively) over 6 years by group. Mean trends are shown as blue lines, 95% confidence intervals are shown in gray

Trends of raw values of EDSS, T25fwt, and 9HPT in Groups A, B, C, and D over 6 years are displayed in Figure 3.

#### 3.4.1 EDSS

Between‐group differences of the average logEDSS were assessed using LMER analyses after correcting for sex, age, and disease duration. Group A had worse average logEDSS compared to Groups B (difference: 0.36 ± 0.10; *p* < .01) and C (difference: 0.48 ± 0.10; *p* < .001), but a similar average logEDSS compared to Group D. The average logEDSS also did not differ between Groups B, C, and D. LogEDSS change over time did not differ between all groups.

#### 3.4.2 T25fwt

Between‐group differences of the average T25fwt^−1^ were assessed using LMER analyses after correcting for age and disease duration. Group A had worse average T25fwt^−1^ compared to all other groups (differences vs.: Group B—0.09 ± 0.02; *p* < .001; Group C—0.10 ± 0.02; *p* < .001; Group D—0.07 ± 0.03; *p* < .05). The average T25fwt^−1^ did not differ between Groups B, C, and D. Moreover, T25fwt^−1^ worsening over time was faster for Group A compared to all other groups (differences vs.: Group B—0.019 ± 0.005/year, *p* < .001; Group C—0.019 ± 0.005/year, *p* < .001; Group D—0.019 ± 0.007/year, *p* < .05). T25fwt^−1^ changes over time did not differ between Groups B, C, and D.

#### 3.4.3 9HPT

Between‐group differences of the average logD9HPT were assessed using LMER analyses after correcting for sex and age. Group A had worse average logD9HPT compared to Groups B (difference: 0.36 ± 0.9; *p* < .001) and C (difference: 0.46 ± 0.9; *p* < .001), but a similar average logD9HPT compared to Group D. The average logD9HPT did not differ between Groups B, C, and D. Moreover, the logD9HPT worsening over time was faster for Group A compared to Group B (difference: 0.06 ± 0.02/year; *p* < .05) and Group C (difference: 0.07 ± 0.02/year; *p* < .05), but similar compared to Group C. Changes of logD9HPT over time did not differ between Groups B, C, and D.

Between‐group differences of the average logND9HPT were assessed using LMER analyses after correcting for sex, age, and disease duration. Group A had worse average logND9HPT compared to all other groups (differences vs.: Group B: 0.54 ± 0.10, *p* < .001, Group C: 0.65 ± 0.10, *p* < .001; Group D: 0.52 ± 0.14, *p* < .01). The average logND9HPT did not differ between Groups B, C, and D. Moreover, the logND9HPT worsening over time was faster for Group A compared to all other groups (differences vs.: Group B: 0.10 ± 0.02/year, *p* < .001, Group C: 0.12 ± 0.02/year, *p* < .001; Group D: 0.11 ± 0.03/year, *p* < .01). LogND9HPT worsening over time was similar between GROUPs B, C, and D.

#### 3.4.4 Relapses

The mean annual number of relapses was higher for Group B (0.45 ± 0.57 relapses/year) compared to Group C (0.24 ± 0.36 relapses/year) (*p* < .05), but did not differ between all other groups (Group A: 0.42 ± 0.63 relapses/year; Group D: 0.22 ± 0.28 relapses/year).

#### 3.4.5 Disease progression

In our Cox analysis no differences between Groups A, B, C, and D were found in terms of disease progression and time‐to‐disease progression.

### 3.5 sNfL across groups

**FIGURE 4:**
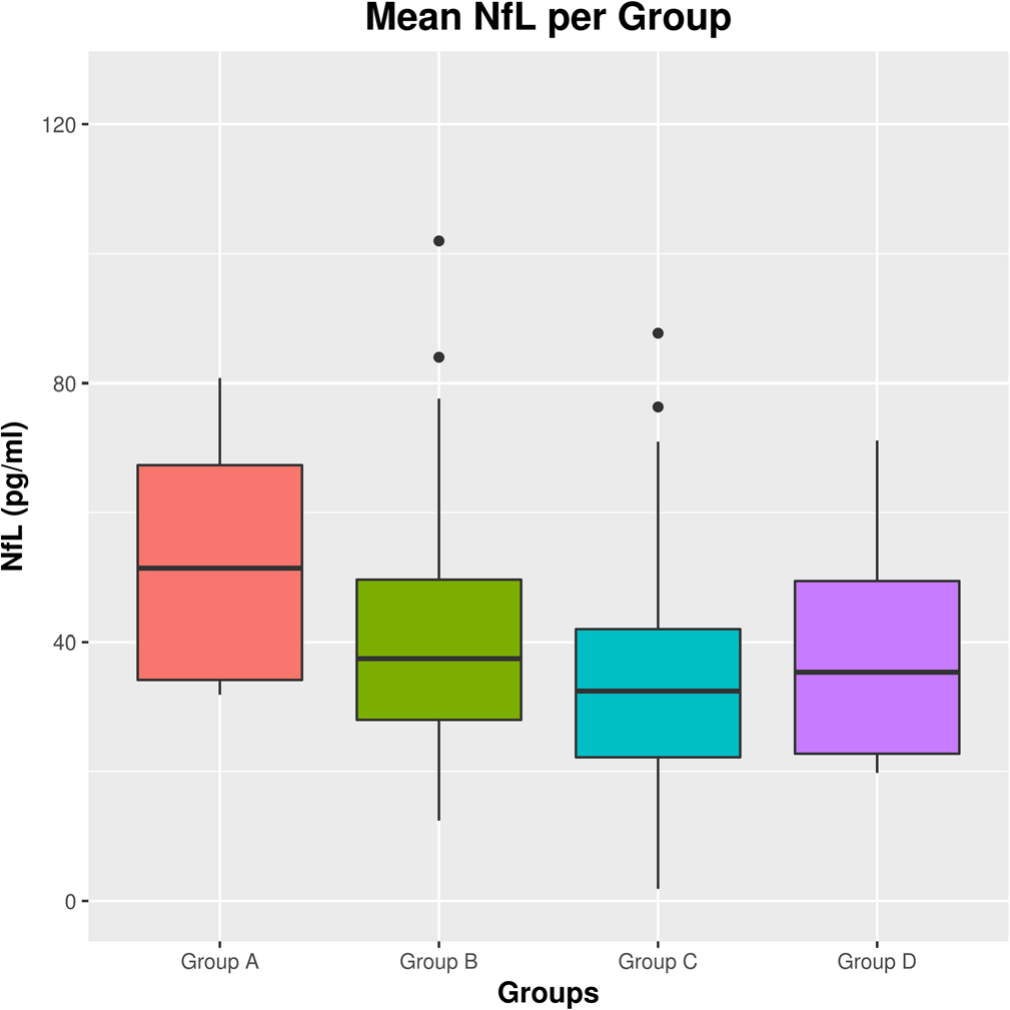
Boxplots of mean serum neurofilament light chain (NfL) levels by group. Whiskers correspond to NfL 25th and 75th percentiles

After correcting for age, mean sNfL of all available time points was higher for Group A (51.4 ± 27.5 pg/mL) compared to all other groups (Group B: 40.2 ± 17.7 pg/mL, *p* < .001; Group C 33.7 ± 14.5 pg/mL, *p* < .001; Group D: 38.5 ± 18.2 pg/mL, *p* < .01). Group B had a trend to higher mean sNfL compared to Group C (*p* = .066). All other between‐group differences were not statistically significant. Raw mean serum sNfL‐values between groups are shown in Figure 4.

## 4 DISCUSSION

In this work, we aimed to reveal distinct anatomic patterns of VLOT in selected CNS structures and then use these patterns to identify patient subgroups in MS. For this purpose, spinal cord, striatal, pallidal, thalamic, cortical gray matter, and white matter volumes were measured in a large MS cohort followed‐up annually over 6 years. Patterns of VLOT were extracted using a principal component analysis after removing the effect of age from the VLOT for each individual CNS structure. These patterns were then entered in a hierarchical clustering analysis for unsupervised identification of patient groups. With this method, we identified four patient groups with distinct anatomic patterns of CNS VLOT. Moreover, we showed that these groups were associated with distinct clinical, radiological, and blood biomarker characteristics.

Our statistical approach allowed to extract four distinct patterns of VLOT. Group A was characterized by a more prominent spinothalamic VLOT compared to all other measured structures. In contrast, Group B displayed a marked pallidal VLOT. Group C was generally characterized by low volume changes and generally low between‐structure VLOT differences. Finally, Group D showed an increase in corticospinal volume as well as a pronounced white VLOT compared to other CNS structures. Patients in these groups did not differ by sex, age, disease duration, and more importantly clinical phenotype. All three clinical phenotypes (relapsing–remitting, secondary progressive, and primary progressive MS) were represented in each of these groups. The 10,000‐fold cross‐validation analysis showed good agreement between the resulting clusters, supporting the robustness of our results in this MS‐population. In summary, our findings clearly add to the evidence currently challenging applied clinical definitions (Kappos et al., 2020; Trapp et al., 2018) and their predictive value.

One patient of our cohort was not classified in either of the four identified subgroups and was excluded from further statistical analysis. Interestingly, this patient was the only pediatric‐onset MS patient of our study. Unfortunately, due to the lack of a larger pediatric‐onset MS group in our cohort, we could not verify if patterns of CNS VLOT differ in this patient group compared to adult‐onset MS patients. Nevertheless, this could potentially be the focus of future investigations.

Interestingly, the four identified groups—resulting from distinct patterns of VLOT across the CNS—displayed differences with regard to their respective clinical courses during the monitoring‐period of our study. Worsening of motor outcomes (walking speed and hand dexterity) was generally more severe and faster in Group A (characterized by pronounced spinothalamic atrophy) compared to all other groups. This finding is in line with recent studies pointing to strong correlations of spinal cord and thalamic volume loss to clinical disease progression (Eshaghi et al., 2018; Magon et al., 2020; Tsagkas et al., 2018b). In accordance to these findings, Group A, compared to all other groups, also had a higher mean sNfL which is a specific biomarker of neuroaxonal damage and loss that also correlates with clinical progression (Barro et al., 2018). EDSS worsening over time (as a continuous variable in our linear mixed effect models) and definite EDSS progression (as binary outcome in our survival analysis) was similar between groups, which may be ascribed to the limitations of the EDSS (e.g., non‐linearity, low sensitivity for clinical changes especially in advanced disease stages) combined with the small number of patients included in some of the identified patient groups. Baseline MS severity score, however, was also higher for Group A, indicating a more aggressive disease course in this group even before the monitoring‐period of our study.

It is worth emphasizing that although average lesion‐load was statistically higher for Group A compared to Groups B and C, T2LV increase over time was similar for all groups. In addition—in contrast to clinical progression, MRI findings, and mean sNfL levels—relapse occurrence did not statistically differ between Group A and the other three groups. Interestingly, Groups B and C differed in terms of relapse rates, even though T2LV increase over time did not differ between groups. Hence, the aforementioned longitudinal volume differences cannot be ascribed to focal inflammatory events—at least not to those occurring in the cerebral white matter. Apart from focal inflammatory activity—manifesting as relapses and demonstrated as lesions on MRI—the presence of a diffuse neurodegenerative component in terms of neuronal and axonal loss as well as demyelination is well established in MS (Carassiti et al., 2018; Evangelou, DeLuca, Owens, & Esiri, 2005; Petrova, Carassiti, Altmann, Baker, & Schmierer, 2018). These two pathomechanisms have been also shown to be at least partly independent from each other (Carassiti et al., 2018; Evangelou et al., 2005; Petrova et al., 2018). Our results may further support a dissociation between focal inflammation and neurodegeneration, as patterns of CNS VLOT corresponded to clinical differences independently of lesion load, although a contribution of spinal and intracortical/subpial lesions—which were not assessed in this study—cannot be excluded.

A recent histopathologic study by Trapp et al. described a distinct subtype of MS characterized by pronounced demyelination restricted to the spinal cord and cortical gray matter but not affecting the brain white matter in 12 autopsy patients, which they then named myelocortical MS (Trapp et al., 2018). These patients were indistinguishable from patients with typical MS, even in light of all clinical and radiological features including T2w hyperintense white matter areas, which surprisingly did not correspond to demyelinated areas in histological analysis. Moreover, volumetric analysis in the MR‐images of those patients revealed a significantly higher cortical gray matter parenchymal fraction and cortical thickness compared to “typical MS.” Interestingly, our Group D showed a paradoxical expansion of spinal cord and cortical gray matter and faster brain white matter atrophy progression compared to other CNS structures (Figure 2). This was unique in this group compared to the other three groups in our cohort. Although increase of CNS volume is theoretically not to be expected in MS patients (especially when assessed over large periods of time, as in our study), a recent study examining large numbers of patients in a longitudinal fashion has also identified a small proportion of patients with brain volume increase (Andelova et al., 2018). Despite the fact that our study cannot provide sufficient evidence to this end, it could be speculated, that Group D corresponds to the described myelocortical MS.

The majority of current therapeutic strategies in MS have an immunomodulatory effect, which has been measured—for the greater part—in terms of a reduction of relapses and new or enlarging lesions (Tintore, Vidal‐Jordana, & Sastre‐Garriga, 2019). Our work displays a certain deviation from this concept with groups generally having similar characteristics in the “no evident disease activity‐3” framework. In addition, a number of relapsing–remitting patients demonstrating a fast progressing CNS atrophy, and a number of secondary progressive and primary progressive MS patients sharing clinical and radiological features with relapsing–remitting patients. Since evaluation of inflammatory activity guides clinical decisions in current daily practice (Sloane, Mainero, & Kinkel, 2015), it might be important to evaluate the effect and efficacy of existing and future immunomodulatory and neuroprotective agents on these atrophy‐pattern‐based patient groups.

Our work demonstrated specific non‐random patterns of volumetric CNS changes in MS patients. Different physiologic and pathologic mechanisms could have contributed to this. Firstly, the extent of cortical demyelination and presence of organized meningeal B‐cell infiltrates, possibly contributing to subpial and cortical pathology, has been shown to vary among MS patients (Albert, Antel, Brück, & Stadelmann, 2007; Howell et al., 2011; Kooi, Geurts, van Horssen, Bø, & van der Valk, 2009). In addition, the blood–brain barrier displays clear structural and functional heterogeneity in different brain regions (e.g., gray matter, white matter, spinal cord, etc.) (Villabona‐Rueda, Erice, Pardo, & Stins, 2019), which may lead to different patterns of CNS damage across regions due to immune cell infiltration. Finally, microglia shows different molecular patterns and morphologies between MS patients and across CNS regions (Lee, Hamanaka, Lo, & Arai, 2019). Of note, analyses of active lesions and cerebrospinal fluid profiles in MS patients support the notion of a dominating single immune‐effector mechanism in each person (Jarius et al., 2017; Metz et al., 2014). Therefore, differences in our groups may—at least partly—reflect distinct pathophysiologic mechanism. Future research may be able to classify patients using single‐time point biomarkers without the need for long‐term longitudinal metrics.

Some considerations for future studies should be discussed. In this proof‐of‐concept study, we consciously avoided to enter brain lesion‐load into our principal component analysis and subsequently into our hierarchical clustering method in order to classify patients solely based on CNS VLOT patterns. This was also done in order to evaluate, whether the identified groups would differ in terms of lesion‐load changes over time. Future investigations should evaluate any potential contribution of brain lesion‐load or ‐number metrics and their localization to a potential MRI‐based classification of MS patients. In addition, our dataset did not contain spinal cord MR‐images (the upper cervical spinal cord volume was extracted from brain MPRAGE images), so that this metric could not be included in our work. Symptomatic and asymptomatic spinal cord lesions currently have a central role in the correct diagnosis (Brownlee, Swanton, Miszkiel, Miller, & Ciccarelli, 2016; Filippi et al., 2016; Geraldes et al., 2018; Thompson et al., 2018; Tintore et al., 2016), prognosis evaluation (Arrambide et al., 2018; Brownlee et al., 2017; Kantarci et al., 2016; Sombekke et al., 2013) and disease monitoring of MS (Zecca et al., 2016). Furthermore, spinal cord lesions correlate strongly with disability (Brownlee et al., 2017; Kearney et al., 2015; Zecca et al., 2016). Hence, it is important that their contribution in a potential MRI‐based patient classification is evaluated in future work.

Moreover, certain aspects of the statistical approach for detection of VLOT patterns across the CNS should be discussed. One alternative to our principal component analysis for this purpose is an independent component analysis. Both principal and independent component analysis are statistical transformations often used prior to running machine learning algorithms such as classification methods as was done in our work. Although the two methods can be used for similar purposes, they demonstrate different features with regard to the extraction of source signals. Principal component analysis searches for orthogonal directions of greatest dispersion of the data (Wold, Esbensen, & Geladi, 1987), which utilizes the commonality of the individual components in the data to define their respective importance. This allows for easier subsequent exclusion of potentially irrelevant data components. On the other hand, in independent component analysis all components are equally important, since this method identifies signal sources independently from the dispersion of the data by calculating a linear transformation that maximizes a criterion, such as non‐Gaussianity (Rutledge & Jouan‐Rimbaud Bouveresse, 2013; Rutledge & Jouan‐Rimbaud Bouveresse, 2015). This makes the use of the independent component analysis for dimensionality reduction in biological datasets challenging compared to the principal component analysis, and previous studies using this method predefined the number of independent components that were used to identify and subsequently evaluate patterns of cortical atrophy in MS patients (Steenwijk et al., 2016). Another important difference is that independent component analysis retains the original vectors, whereas principal component analysis only retains a linear transformation of them. The corollary of this is that the proportions of independent component analysis can be more easily related to the amount of change due to a single phenomenon, while principal components cannot as they are usually influenced by several phenomena. As such, independent component analysis allows for greater interpretability of the initial data using the initial variables (in our case VLOT in multiple CNS regions) compared to principal component analysis. Therefore, both methods have inherent advantages and disadvantages. Recently, optimization of these methods has been attempted with approaches such as independent principal component and common component analysis (Bouhlel et al., 2018; Rutledge, 2018; Yao, Coquery, & Lê Cao, 2012). Future studies should evaluate, which of these methods delivers optimal results with regard to an objective MRI‐based classification of MS patients using separate training and validation cohorts.

A number of limitations need to be mentioned. Some patients were lost to follow‐up during the study, leading to incomplete data sets and potential biases. Moreover, despite the fact that the examined cohort was relatively large, two of the groups were rather small (Group A: 14 patients; Group D: 11 patients). In addition, because of the insufficient number of patients, we could not validate our results using a training, a validation and a test dataset; therefore, the existence of other potential groups, which could possibly be identified with our method in a larger population, cannot be excluded. For this reason, larger longitudinal multi‐center studies would be required to validate our results in the future. The lack of a control group to compare VLOT between the MS‐groups and healthy subjects is a clear limitation of our study, despite the fact that we accounted for the effects of normal aging in our statistical analyses. For this reason, we were not able to assess differences between patterns of CNS volume loss over time in “healthy” aging and multiple sclerosis. An effect of treatment on the results of our classification cannot be excluded. Indeed, patients of Group A were less commonly treated compared to Group C, whereas the distribution of treatments used in Group C was different compared to Group D. However, it is unclear, if and to which extent this could influence the presence of distinct patterns of VLOT in our patients and consequently our classification results. Nevertheless, the majority of our patients were treated with first‐line injectables, which have been shown to have a negligible effect on CNS‐atrophy (Favaretto, Lazzarotto, Margoni, Poggiali, & Gallo, 2018). Another potential limitation of our longitudinal experimental design relates to potential volumetric measurement variability due to methodological or physiological factors. For the segmentation of cortical gray matter, CIVET inherently co‐registers MR‐images into a halfway space before MRI analysis in order to help “treat” all study participant data equally in the image processing context in order to limit potential experimental biases. The MAGeT brain algorithm used for segmentation of deep gray matter structures accounts for this variability source by generating a template library from the dataset under evaluation, which included MRI datasets from all timepoints as well as representative demographic and clinical characteristics of the whole patient sample. Hence, this approach should mitigate bias of experimental sources across the input data set. Furthermore, with regard to the preprocessing steps of our MRI data prior to the segmentation of brain structures, the MAGeT registration procedure is similar to CIVET, which should at least reduce potential biases between these two algorithms that calculated the cortical, thalamic, pallidal, and striatal volumes used in this study. However, this step differs in SIENAX used for white matter volumes, which could have introduced bias in our volumetric assessment. Finally, despite the fact that our clustering analysis uniquely classified each patient in one group, it cannot be excluded that the identified groups represent different “states,” from or to which individual patients shift during the course of the disease.

In summary, in this study, we identified patient groups with distinct patterns of regional volume change over time across the CNS in a large MS‐cohort. This study suggests that these patterns of longitudinal CNS volume change at least partially correspond to different patterns of clinical progression and sNfL‐levels. Our work also supports the value of MRI‐assessed volume change patterns in the subclassification of MS patients and provides proof of concept for future studies.

**Abbreviations**
D9HPT: dominant hand 9‐hole peg test
EDSS: expanded disability status scale
LMER: linear mixed effect models
MPRAGE: magnetization‐prepared rapid gradient‐echo
ND9HPT: non‐dominant hand 9‐hole peg test
sNfL: serum neurofilament light chain
T25fwt: timed 25‐ft walk test
T2LV: T2‐weighted lesion volume
VLOT: volume loss over time

## CONFLICT OF INTEREST

Charidimos Tsagkas, M. Mallar Chakravarty, Yvonne Naegelin, and Michael Amann have no disclosures.

Simon Pezold received research support from the Novartis Research Foundation.

Katrin Parmar holds a grant of the Baasch‐Medicus foundation. Her institution (University Hospital Basel) received speakers' honoraria from Novartis and ExceMED and travel support by Novartis Switzerland.

Laura Gaetano is an employee of F. Hoffmann‐La Roche Ltd, Basel, Switzerland.

Athina Papadopoulou has consulted for Teva, received speaker‐fee from Sanofi‐Genzyme and travel support from Bayer AG, Teva, UCB‐Pharma AG and Roche. Her research was/is being supported by the University of Basel, the Swiss Multiple Sclerosis Society, the Swiss National Science Foundation and the “Stiftung zur Förderung der gastroenterologischen und allgemeinen klinischen Forschung sowie der medizinischen Bildauswertung.”

Jens Wuerfel: CEO of MIAC AG, Basel, Switzerland; speaker honoraria (Bayer, Biogen, Novartis, Teva); advisory boards and research grants (Biogen, Novartis, La Roche, Sanofi); supported by the German Ministry of Science (BMBF/KKNMS) and German Ministry of Economy (BMWi).

Ludwig Kappos' institution (University Hospital Basel) has received research support and payments that were used exclusively for research support for Dr Kappos' activities as principal investigator and member or chair of planning and steering committees or advisory boards in trials sponsored by Actelion, Addex, Almirall, Bayer HealthCare, Celgene, CLC Behring, Genentech, GeNeuro, Genzyme, Merck Serono, Mitsubishi Pharma, Novartis, Octapharma, Ono, Pfizer, Receptos, F. Hoffmann‐La Roche, Sanofi‐Aventis, Santhera, Siemens, Teva, UCB, and XenoPort; license fees for Neurostatus 4 products; research grants from the Swiss Multiple Sclerosis Society, the Swiss National Science Foundation, Innosuisse, the European Union, and the Roche Research Foundation.

The current (DKD Helios Klinik Wiesbaden) or previous (University Hospital Basel) institutions of Till Sprenger have received payments for speaking or consultation from: Biogen Idec, Eli Lilly, Allergan, Actelion, ATI, Mitsubishi Pharma, Novartis, Genzyme and Teva. Dr. Sprenger received research grants from the Swiss MS Society, Novartis Pharmaceuticals Switzerland, EFIC‐Grünenthal grant, and Swiss National Science Foundation.

Dr. Kuhle reports grants from Biogen, grants from Novartis, grants from Roche, grants from Swiss MS Society, grants from Sanofi, grants from University of Basel, grants from Swiss National Research Foundation, grants from Merck, grants from Celgene, grants from Progressive MS Alliance, outside the submitted work.

Stefano Magon is an employee of F. Hoffmann‐La Roche Ltd, Basel, Switzerland.

## ETHICS APPROVAL STATEMENT

The local ethics committee approved the study (EKBB‐46/04).

## PATIENT CONSENT STATEMENT

All patients provided written informed consent.

## Supporting information


**TABLE S1** Correlation matrix between the residuals of the mean annual atrophy rates of multiple sclerosis patients after correcting for age

## Data Availability

Anonymized raw data are available upon reasonable request.
