# Supplementary material for: Classification of multiple sclerosis based on patterns of CNS regional atrophy covariance
Source: Hum Brain Mapp. 2021 Feb 24;42(8):2399–415. doi: 10.1002/hbm.25375 (PMC8090784; doi:10.1002/hbm.25375)
Supplement: Supplementary file 1 — TABLE S1 Correlation matrix between the residuals of the mean annual atrophy rates of multiple sclerosis patients after correcting for age [file HBM-42-2399-s001.docx]

**Supplementary Material Table 1** Correlation matrix between the residuals of the mean annual atrophy rates of multiple sclerosis patients after correcting for age.

|  | SC | Striatum | Globus Pallidus | Thalamus | Cortical GM | Brain WM |
| --- | --- | --- | --- | --- | --- | --- |
| SC | 1.00 | 0.24 | 0.04 | 0.47 | 0.32 | 0.07 |
| Striatum | 0.24 | 1.00 | 0.44 | 0.55 | 0.16 | 0.52 |
| Globus Pallidus | 0.04 | 0.44 | 1.00 | 0.18 | 0.19 | 0.37 |
| Thalamus | 0.47 | 0.55 | 0.18 | 1.00 | 0.40 | 0.25 |
| Cortical GM | 0.32 | 0.16 | 0.19 | 0.40 | 1.00 | -0.32 |
| Brain WM | 0.07 | 0.52 | 0.37 | 0.25 | -0.32 | 1.00 |

SC = spinal cord, GM = grey matter, WM = white matter

All correlations have been calculated as Pearson’s correlation coefficients on the residuals of the regression analysis between the atrophy rate of each region and age.
